# Efficacy of curcumin for amelioration of radiotherapy-induced oral mucositis: a preliminary randomized controlled clinical trial

**DOI:** 10.1186/s12885-023-10730-8

**Published:** 2023-04-17

**Authors:** Vahid Ramezani, Shiva Ghadirian, Masoud Shabani, Mohammad Ali Boroumand, Reza Daneshvar, Fatemeh Saghafi

**Affiliations:** 1 Department of Pharmaceutics, School of Pharmacy, Shahid Sadoughi University of Medical Sciences and health services, Yazd, Iran; 2 Pharmaceutical Sciences Research Center, School of Pharmacy, Shahid Sadoughi University of Medical Sciences and health services, Yazd, Iran; 3 Department of Radiation Oncology, School of Medicine, Shahid Sadoughi University of Medical Sciences and health services, Yazd, Iran; 4 Department of Clinical Pharmacy, School of Pharmacy, Shahid Sadoughi University of Medical Sciences and health services, Shohadaye gomnam Blvd., Yazd Province, Yazd, Iran

**Keywords:** Radiotherapy induced Mucositis, Neoplasms, Curcumin

## Abstract

**Background:**

Oral mucositis (OM) is one of the main problems in almost all patients undergoing head and neck radiotherapy (RT). Owning to the antioxidant and anti-inflammatory properties of curcumin, the effect of both oral and topical formulations of curcumin was assessed on radiation-induced OM (ROM) in this study.

**Methods:**

The safety and efficacy of curcumin mouthwash 0.1% (w/v) and curcumin-nanocapsule were evaluated in ameliorating severity and pain/burning associated with OM during RT. The current randomized, placebo-controlled trial was conducted on 37 patients with head and neck cancers. Patients with grades 1 to 3 of ROM were randomized to receive one of the three interventions: curcumin mouthwash (0.1% w/v); Sinacurcumin soft gel containing 40 mg curcuminoids as nano-micelles (SinaCurcumin®40); or placebo mouthwash with a similar transparent appearance to curcumin mouthwash for 1 min three times daily during RT. Study evaluations were conducted at baseline and weekly thereafter for up to 3 weeks using the Numeric rating scale (NRS) and world health organization (WHO) scale.

**Results:**

Among the 45 patients randomized, 37 (mean (SD) age of 53.36 (15.99) years; 14 [37.8%] women) completed the treatment according to the protocol. Patients treated with either oral or topical curcumin showed a significantly reduced severity and burning related to OM during the first 3 weeks after administration (P-Value < 0.001) as compared with the placebo. At study termination, more than 33% of subjects utilizing curcumin mouthwash and 15% of patients utilizing curcumin-nanocapsule remained ulcer free while all of the placebo-receiving subjects had OM. The reduction of NRS and WHO scale between curcumin groups was comparable without significant differences.

**Conclusion:**

Both curcumin mouthwash and nanocapsule were effective, safe, and well-tolerated in the treatment of radiation-induced OM. Higher doses of curcumin and larger sample sizes can be used for further investigation in future studies.

**Trial registration:**

https://irct.ir/ IRCT20190810044500N17 (13/08/2021).

## Background

RT is a common and effective treatment modality for various types of cancer [1]. This approach is used in almost 60% of cancer patients alone or in combination with other treatments such as surgery and chemotherapy to treat, prevent, and alleviate the complication [2]. The general principle in RT is to deliver the right dose of radiation to the affected or endangered tissues with minimal exposure for the normal tissues. This can be achieved with the help of new RT techniques using multiple fields, at different angles and appropriate depths and energies.

Oral complications of RT in the head and neck include inflammation and swelling of the mucous membranes and skin, periodontal infection, fungal and viral infections, and salivary gland disorders [3, 4]. Inflammation of the oral mucosa or mucositis is one of the main problems in almost all patients undergoing head and neck radiotherapy, leading to poor quality of life, reduced quality of nutrition, and pain in the mouth. It also increases the risk of infection in patients. The severity of the problem increases with the continuation of treatment, to the point that the pain and burning sensation sometimes cause the patient to stop continuing treatment [5, 6].

These lesions are due to the local effect of ionizing radiation on the oral mucosa and differ from one patient to another depending on oral hygiene, radiation levels, specific diseases, and smoking [7]. During RT, subcutaneous tissue is inevitably exposed to radiation, leading to several unpleasant acute or chronic skin complications and skin reactions ranging from acute erythema to necrosis [8]. More than 90% of RT-treated patients experience some degree of skin reaction during or shortly after the treatment [2, 8, 9]. The radiation-induced damage to a tumor or normal tissue is mainly the result of indirect mechanisms involving free radicals. Highly proliferating tissues such as the epithelium of the skin and mucosa are the most affected areas by radiation [9]. The direct mechanism occurs through DNA ionization [10].

So far, various methods have been introduced for the prevention and treatment of mucositis (e.g. laser and cryotherapy, topical medications, antibiotics and antiseptics, antifungals, anti-inflammatory agents, cellular protectors, immunosuppressant, anesthetics, and use of normal saline mouthwashes, baking soda, betadine, and Chlorhexidine); however, none of these methods managed to completely reduce mucositis [11–14].

Curcumin, a diferuloylmethane, is a phenolic compound. The active ingredient of the rhizome of the plant is turmeric which makes up about 2 to 8% of turmeric compounds [15, 16]. Curcumin is one of the most important plant-based compounds, which has been long utilized for the treatment of problems in the respiratory system, liver, sinusitis, and gastrointestinal tract, wound healing, and pain/burning relief. Studies have shown that curcumin has pharmacological activities including antioxidants, inhibition of inflammatory factors, induction of cell death, anticarcinogen, and inhibition of intracellular pathways in disease [17, 18].

Curcumin interferes with inflammatory responses can reduce the activity of cyclooxygenase-2, lipoxygenase, and nitric oxide synthetase, and inhibit the production of inflammatory cytokines such as alpha tumor necrosis factor (TNF-alpha) [19–21]. It also eliminates free radicals within the tissues and prevents their damage [22]. It also protects normal cells against carcinogenesis. This compound shows its antioxidant effects by inhibiting superoxide radicals, hydrogen peroxide, and nitric oxide. It has been shown also to regulate the cellular activity of cytokines [23, 24]. Curcumin has been used to treat skin conditions such as itching, acne, eczema, and wrinkled skin as well as wound healing [25].

Curcumin may improve morphine function at high doses by suppressing pain transmission pathways. These effects are due to analgesic chemical mediators such as serotonin, dopamine, and noradrenaline, whose release was increased by curcumin to reduce pain [26]. Curcumin increases the level of chemical mediators by increasing the number of presynaptic terminals of this neurotransmitter and increasing their opening time [27]. It also increments the level of 5-hydroxytryptophan (a precursor of serotonin) and the sensitivity of postsynaptic cells to this substance, leading to an increase in serotonin levels [28]. Curcumin also enhances the life of these neurotransmitters and their effects by inhibiting the enzyme monoamine oxidase B and A (enzymes that break down dopamine and serotonin in the synaptic space) [36].

Various medicinal products are currently available to relieve and treat the RT-related damage in cancer patients. In addition to their therapeutic properties, these chemicals suffer from their own side effects. The use of herbal medicines is recommended to prevent and increase tissue resistance and ultimately reduce the severity of RT-induced injuries [29]. Although the effect of curcumin is evaluated in different studies [30, 31], it is not currently accepted or recommended to prevent or treat ROM in the guidelines of the Multinational Association of Supportive Care in Cancer and International Society of Oral Oncology (MASCC/ISOO) [32, 33]. Considering the appropriate antioxidant and anti-inflammatory properties of curcumin, this study aimed to assess the effect of both oral and topical formulations of curcumin on ROM.

## Materials and methods

This prospective single-blind randomized placebo-controlled clinical trial was performed at Ramezanzadeh Radiotherapy Center in Yazd, Iran from September 2020 to May 2021. Following ethical approval from the Ethics Committee of Shahid Sadoughi University of Medical Sciences (ID: IR.SSU.MEDICINE.REC.1399.112), 45 patients who developed RT-induced oral inflammation were included and randomly divided into three groups. This study was also registered in the Iranian Registry of Clinical Trials (IRCT) with the registration ID of IRCT: IRCT20190810044500N17 (13/08/2021). The participants were asked to sign a written informed consent form. All experiments were performed in accordance with relevant guidelines and regulations.

### Curcumin mouthwash preparation

To prepare curcumin mouthwash, 100 mg of curcumin powder (Karen, Iran) was first dissolved in 20 mL of polyethylene glycol 400 (Merck, Germany) under overnight stirring at 60 °C. Then, 158 mg of sodium hydroxide and 680 mg of KH_2_PO_4_ (Merck, Germany) were dissolved in 100 ml of water to prepare the buffer with a pH of 7.4. Then, 12 mg of sodium saccharin was dissolved in 80mL of the buffer as a sweetener and added to curcumin-containing polyethylene glycol at 25 °C and stirred until complete dissolution at 250 rpm (Heidolph MR-Hei Standard Hot Plate Magnetic Stirrer). Subsequently, 10 mg of menthol was added to the formulation as a flavoring agent after dissolving in 1ml of polyethylene glycol 400. The mouthwashes were daily prepared and kept in the refrigerator with no microbial preservatives to prevent their side effects. The placebo formulation contained sunset yellow as a colorant as well as all the materials used in the curcumin mouthwash except the active ingredient. Microbial contamination of both formulations was controlled by culturing on Mueller Hinton agar, soybean casein digest agar, and Sabouraud dextrose agar which revealed no microbial pollution.

### Participants and study procedure

This study was designed as a 3-group, single-blind, placebo-controlled phase II preliminary randomized clinical trial. The assessor of clinical symptoms was blinded to the intervention assignments during the study. Inclusion criteria were as follows: Patients with mild to moderate ROM (Grade 1 to 3) over the age of 18 who received the minimum radiation dose of 50 g during the entire study. In addition, patients with the following conditions were removed from the study: uncontrolled underlying systemic disease, smoking, serious dental problems, receiving other concomitant treatments for OM, simultaneous treatment of chemotherapeutic drugs, OM severity rated 4 on a 1-to-4 WHO scale and active infection.

Forty-five eligible participants were registered and randomized using a computer-generated random list in a 1:1:1 ratio to receive one of the three interventions: curcumin mouthwash (0.1% w/v); Sinacurcumin soft gel containing 40 mg curcuminoids as nano-micelles (SinaCurcumin®40); or placebo mouthwash with a similar transparent appearance to curcumin mouthwash. Sinacurcumin soft gel (SinaCurcumin®40) was purchased from Exir Nano Sina Pharmaceutical Company. The treatment protocol was initiated within 48 h of enrollment. All the patients were asked to use 10 ml of freshly prepared mouthwashes 3 times a day or one capsule of SinaCurcumin®40 once a day for up to 21 days.

The severity of OM was assessed using the WHO Oral Toxicity Scale [34], scored on a 4-grade scale (1, soreness ± erythema; 2, erythema and ulcer, but patient can swallow solid foods; 3, ulcers with extensive erythema and the patient can not swallow food; and 4, mucositis to the extent that alimentation is not possible). NRS was used to assess treatment response in terms of pain level or burning mouth, which ranged from 0 (no burning) to 10 (severe burning). Patients were asked to use acetaminophen as needed if RT related pain or burning was persecutor or intolerable. The severities of OM and OM-related burning were considered the primary and secondary outcomes, respectively. Patients were evaluated at baseline and followed up at weekly intervals thereafter until the termination of the study (21 days).

### Sample size and statistical analysis

This RCT pilot study was developed to calculate the sample size for a larger RCT. Considering the rule of thumb for the pilot studies, 12 participants in each group would be an appropriate justification for sample size [35]. The obtained data were analyzed using SPSS version 25.0 software (SPSS Inc.). Chi-square and Fisher’s exact tests were considered to compare qualitative variables. Thereafter, the Kruskal Wallis test and Wilcoxon sign rank were employed to compare the changes in pain/burning scores and OM grades during the 3-week follow-up. One-way and two-way analyses of variance tests were used along with a Bonferroni-adjusted post hoc test or its non-parametric equivalent if necessary. Finally, a repeated measurement test was used to compare the changes in the quantitative variables over time. The significance level was considered P-value < 0.05 for all study variables.

## Results

**Fig. 1:**
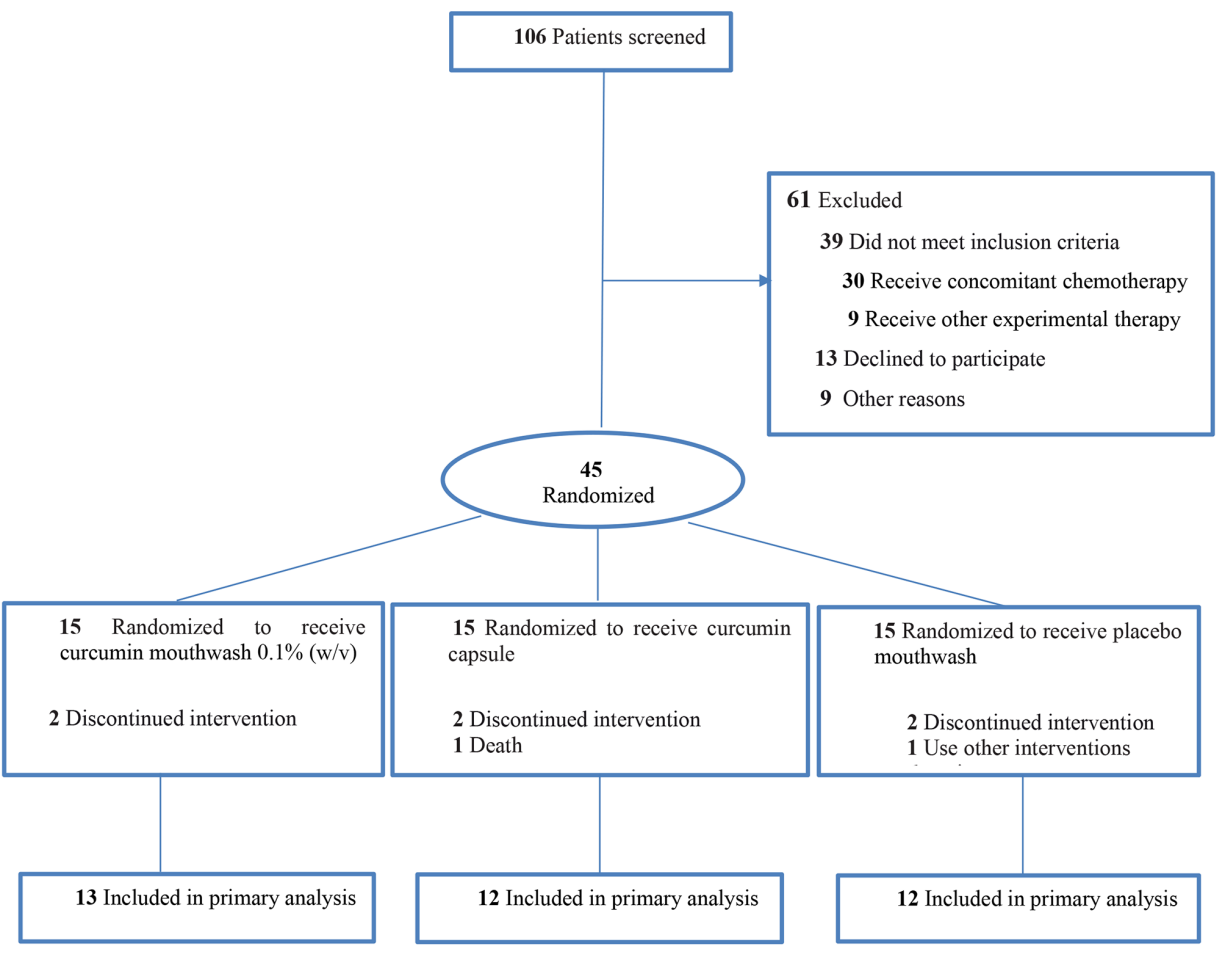
Recruitment, Randomization, and Patient Flow to Treat Oral Mucositis

Among the 106 patients screened over the study period, 61 patients declined to participate or were found ineligible before starting the treatment, thus, they were excluded. During the follow-up, nine patients (2 in the curcumin mouthwash group, 3 in the curcumin capsule group, and 4 in the placebo mouthwash group) were excluded from the primary analysis (Fig. 1). Finally, 37 patients, 23 men and 14 women with a mean (SD) age of 53.36 (15.99) years completed the study. The baseline characteristics of the patients are listed in Table 1.

### Primary and secondary outcomes

**Fig. 2:**
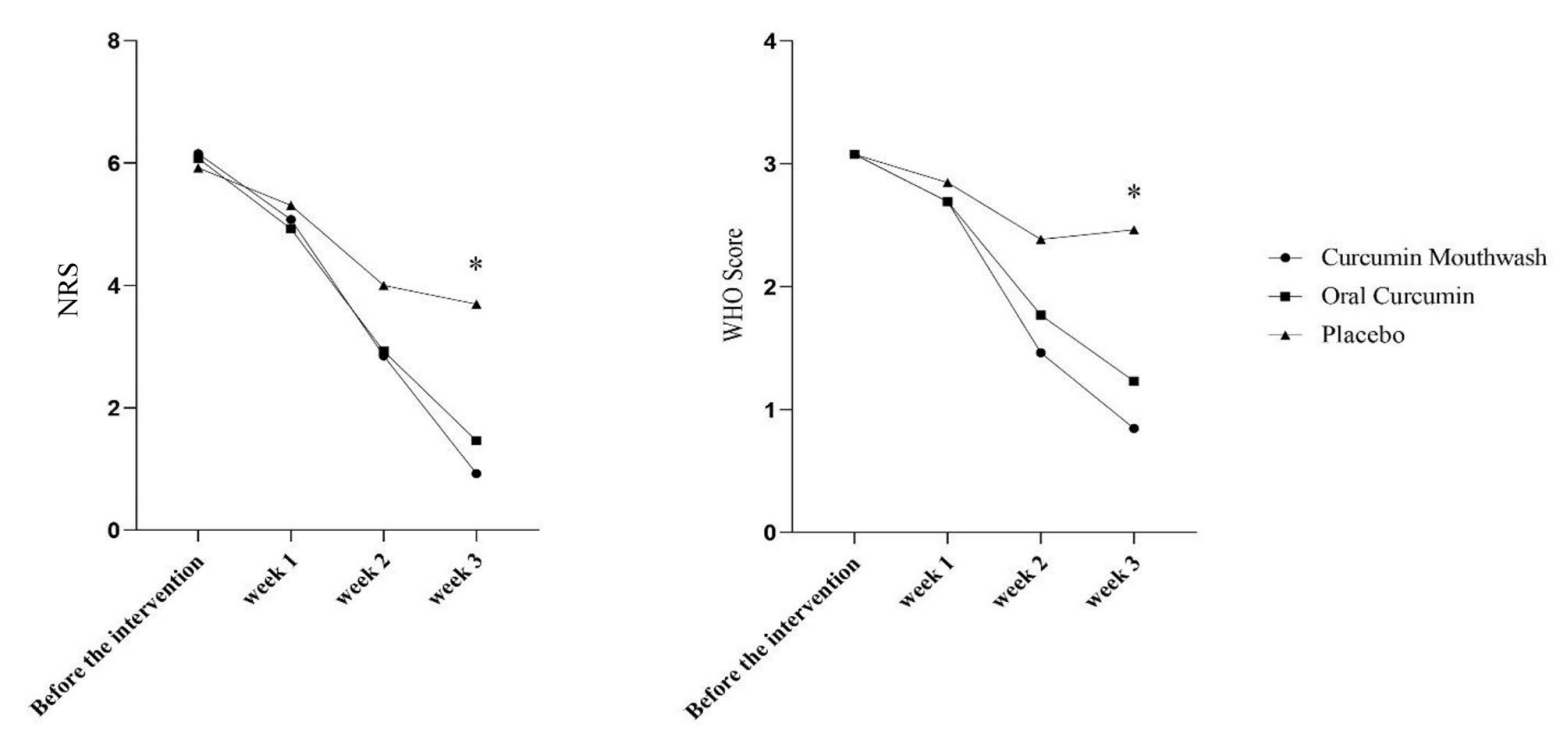
Variation of the primary and secondary outcomes over time; *P-value < 0.05 considered statistically significant

At study termination, more than 33% and 15% of curcumin mouthwash and curcumin-nanocapsule-receiving subjects remained ulcer free compared with none of the placebo-treated subjects, respectively. The means (SD) of WHO and NRSscales did not significantly differ between the three groups over the weeks 1 and 2 while Both WHO and NRS scales in each of the curcumin-treated groups were significantly reduced at the study periods of week 3 compared to the placebo group (P < 0.001) (Fig. 2).

**Table 1:** Patient Demographics Profile and Baseline Disease Characteristics

Characteristic		Curcumin Mouthwash N = 13	Curcumin capsule N = 12	Placebo mauthwash N = 12	P-Value
Sex, N %)	Men	8 (61.5)	7 (58.4)	8 (66.6)	0.95
	Women	5 (38.5)	5 (41.6)	4 (33.4)	
Age, mean ± SD (y)		51.85 (16.94)	55.7 (17.07)	52.5 (14.83)	0.84
Radiotherapy session, mean (SD)		7.23 (2.04)	7.33 (2.3)	6.8 (2.7)	0.82
Type of malignancy, N (%)	Thyroid	1 (7.6)	1 (8.3)	1 (8.3)	0.61
	Nasopharyngeal	2 (15.4)	1 (8.3)	0 (0.0)	
	Orophareyngeal	2 (40)	3 (25.0)	0 (0.0)	
	Larynx	1 (7.6)	2 (16.6)	1 (8.3)	
	Neck	2 (33.3)	1 (8.3)	3 (25.0)	
	Tongue gland	2 (15.4)	1 (8.3)	1 (8.3)	
	Mouth	1 (7.6)	2 (16.6)	3 (25.0)	
	Salivari gland	0 (0.0)	1 (8.3)	2 (16.6)	
	Maxillofacial	2 (15.4)	0 (0.0)	0 (0.0)	
PMH, N (%)	Diabates mellitus	3 (23.1)	1 (8.3)	2 (16.6)	0.45
	Dislipidemia	0 (0.0)	3 (25.0)	0 (0.0)	
	Blood pressure	2 (15.4)	0 (0.0)	2 (16.6)	
	Anemia	2 (15.4)	1 (8.3)	0 (0.0)	
	Hypothyroidism	0 (0.0)	2 (16.6)	1 (8.3)	
	BPH	1 (7.6)	0 (0.0)	1 (8.3)	
	Cardiovascular Diseases	1 (7.6)	1 (8.3)	2 (16.6)	
	Respiratory Disease	1 (7.6)	2 (16.6)	0 (0.0)	
	Renal Disease	1 (7.6)	1 (8.3)	1 (8.3)	
Concurrent medication, N (%)	Antihypertensive drugs	2 (15.4)	0 (0.0)	2 (16.6)	0.70
	Antidiabetic drugs	3 (23.1)	1 (8.3)	2 (16.6)	
	Antiplatelet agents	1 (7.6)	1 (8.3)	2 (16.6)	
	Others	5 (38.5)	8 (50)	3 (25.0)	
Smoking, N (%)		3 (23.1)	3 (25.0)	4 (33.3)	0.74
History of addiction, N (%)		2 (15.4)	1 (8.3)	1 (8.3)	0.82
Educational status, N (%)	Illiterate	1 (7.6)	1 (8.3)	0 (0.0)	0.83
	Primary school	1 (7.6)	1 (8.3)	1 (8.3)	
	Secondary school	1 (7.6)	3 (25.0)	0 (0.0)	
	Diploma	5 (38.5)	1 (8.3)	4 (33.3)	
	BA	3 (23.1)	4 (33.3)	3 (25.0)	
	BS	1 (7.6)	1 (8.3)	2 (16.6)	
	MS	1 (7.6)	1 (8.3)	1 (8.3)	

N: Number; SD: Standard Deviation; y: yaer; BA: Bachelor of Art; BS: Bachelor of Science; MS: Master of Science; BPH: Benign Prostatic Hyperplasia.

Repeated measurement analysis compared the reduction of WHO scale at weeks 1 to 3 relative to baseline. The results indicated a statistically significant decline in the oral or topical curcumin groups vs. the placebo group. Additional analysis showed a significant reduction in NRS in both curcumin groups compared with the placebo group except for weeks 3 vs. 2 (P-Value = 0.81) and week 1 vs. baseline (P-Value = 0.29) in the control group (Table 2). No significant intra-group differences were observed in the overall incidence of adverse symptoms.

**Table 2:** Primary and secondary outcomes over the time

Variables	Comparing		Curcumin Mouthwash		Curcumin capsule		Placebo Mouthwash		Between group P-Value
			Mean Difference	Within group P-Value	Mean Difference	Within group P-Value	Mean Difference	Within group P-Value	
**WHO scale**	W0	W1	0.385	0.018	0.33	0.20	0.36	0.100	0.981
		W2	1.61	0.001	1.16	0.001	0.92	0.051	0.150
		W3	2.23	0.001	1.70	0.001	0.72	0.070	0.002
	W1	W2	1.23	0.001	0.83	0.001	0.54	0.052	0.074
		W3	1.84	0.001	1.40	0.001	0.36	0.340	< 0.001
	W2	W3	0.60	0.014	0.58	0.027	0.18	0.441	0.029
**NRS**	W0	W1	1.00	0.001	1.25	0.001	0.455	0.29	P < 0.01
		W2	3.20	0.001	3.25	0.001	1.63	0.001	P < 0.01
		W3	5.10	0.001	4.58	0.001	1.72	0.005	P < 0.01
	W1	W2	2.23	0.001	2.00	0.001	1.18	0.019	P < 0.01
		W3	4.10	0.001	3.33	0.001	1.27	0.002	P < 0.01
	W2	W3	1.90	0.036	1.33	0.008	0.09	0.81	P < 0.01

## Discussion

In the current single-blind, randomized clinical trial, patients treated with either the oral or topical curcumin showed a significantly reduced severity and burning related to OM during the first 3 weeks after administration as compared with the placebo group. However, no difference was observed between oral and topical curcumin groups.

Determination of the appropriate radiation sensitivity is determinantal in increasing the chance of tumor cell death and minimizing the radiation damage to normal tissues [36]. Studies have shown that curcumin can effectively treat dermatitis, pneumonia, cataracts, secondary tumors, and mucositis/enteritis. Many of these diseases are inflammatory in nature, so it can be concluded that the anti-inflammatory effects of curcumin can reduce the inflammatory diseases, possibly by reducing the production of inflammatory molecules as well as contributing to maintaining the antioxidant-oxidant balance [37–41].

In 2016, Elad et al. reported that curcumin mouthwash is safe and tolerable for the prevention of mucositis in children [42]. The bioequivalence of synthetic versus natural curcumin was shown in an *in-vitro* assay [43]. As the bioavailability of curcumin is poor, it does not appear to be toxic even at high doses [30]. Previous studies showed that curcumin had positive therapeutic effects in reducing radiotherapy-induced acute and chronic dermatitis in mice, which was associated with decreased levels of proinflammatory cytokines and fibrogenic cytokines such as TGF-β [44, 45]. Ryan et al. showed that the efficiency of the treatment of radiotherapy-induced dermatitis by curcumin is associated with a reduction in scaling in the curcumin group, but there was not much difference in the pain scores reported by the patient [46].

In a single-blind, randomized trial, Rao et al. treated the RT-receiving patients with iodine/pharyngeal neoplasms, or povidone-iodine and curcumin. Although the incidence of total mucositis did not differ between groups, the curcumin group experienced less intolerable mucositis as well as decreased mucosal severity. It should be considered that povidone-iodine is uncommonly used for ROM. Similar common agents such as lidocaine and chlorhexidine have no effect because they are designed for symptomatic treatment while curcumin is focused on reducing molecular inflammatory factors [47]. In the current study patients who received either oral or topical curcumin experienced less intolerable mucositis.

Almost all of the studies evaluating the anti-inflammatory effect of curcumin showed satisfactory outcomes. Mansourian et al. compared the effect of curcuma longa or placebo topical gel for 8 weeks on ROM. They revealed that patients who received curcuma longa experienced lower OM grades and milder burning mouth sensation. In our study patients in curcumin groups reported feeling more comfort in their mouth [30]. The results of another trial by Thomas et al. showed 0.1% triamcinolone acetonide oral paste reduced burning sensation more than curcumin oral gel (61). The protective effect of curcumin on chemotherapy-induced intestinal disorders has been also shown by Yao et al. [48]. Evaluating the effect of curcumin mouthwash in mucositis patients due to RT and chemotherapy indicated the better effects of curcumin on wound treatment and control of side effects compared with chlorhexidine [49]. Another study revealed a significantly lower severity of OM in all examination sessions of the experimental group compared to the controls. Curcumin did not cause any oral side effects. In the end, they concluded that curcumin was effective in delaying the appearance of OM and reducing its severity [50].

In a clinical trial study, Rayan et al. reported that oral curcumin reduced the severity of dermatitis in breast cancer patients compared with the placebo group (P-Value = 0.008) during radiation therapy [26]. In the present study, after three weeks of treatment with curcumin, the rate of improvement of mucositis and oral inflammation (examined by the WHO criteria) was significantly increased. These results are in line with previous studies expressing the antioxidant and anti-inflammatory properties of curcumin.

The results of three recent systematic reviews suggested curcumin is safe, remarkably well tolerated, and effective in preventing and ameliorating of mucositis lesions. As the number of the included studies were few, all of them recommended that further well-designed investigations is required to confirm the promising effects of curcumin in mucositis lesions [51–53]. Results of the current study showed positive effects of both oral and topical curcumin on reducing pain and burning, which can be due to the effect of curcumin on the nerve pathways as well as the accelerating the healing of oral inflammation.

Up to now, many studies have been conducted to reveal the protective effects of curcumin against pain and burning [30, 47]. Curcumin increases noradrenaline levels in the frontal lobe and hippocampus of the brain, which is effective in reducing pain [54]. Various studies have confirmed the ability of curcumin to affect opioid receptors and its effects on pain and relief of superficial pain through the opioid system [55]. Curcumin has been shown to inhibit chemical mediators of inflammation, such as cyclooxygenase 2. Cyclooxygenase 2 causes the synthesis of prostaglandins (mediates inflammation and fever) and curcumin reduces the inflammatory process by inhibiting this pathway [56]. PKC (protein kinase C) is a substance that increases the expression of cyclooxygenase 2. Inhibition of cyclooxygenase 2 and nitric oxide by curcumin is achieved through the action of nuclear factor kappa B [57]. Furthermore, curcumin inhibits the metabolism of arachidonic acid, leading to the production of lipoxygenase and cyclooxygenase [58].

This study, however, had several limitations. Although 106 patients were screened for eligibility criteria, only a limited number of them were eligible and completed the study due to the COVID-19 pandemic. Further studies with a larger sample size are recommended to establish the role of topical or oral curcumin in ROM management. Other confounding factors included radiation site and dose, which could affect the severity of OM. Future studies should address the potential effect of oral or topical curcumin in a dose-finding study.

## Conclusion

Thanks to its anti-inflammatory nature, curcumin can be effective in improving inflammatory lesions of the mouth caused by radiotherapy to the head and neck. Curcumin in each of oral or topical formulations was significantly effective in reducing pain and burning related to ROM. The oral or topical forms of curcumin, however, showed no significant difference. Studies addressing higher doses of curcumin in larger sample sizes are suggested in future studies.

## Data Availability

All data generated or analyzed during this study are included in this published article.

## List of abbreviations

OM: Oral Mucositis
ROM: Radiation-induced Oral Mucositis
RT: Radiotherapy
NRS: Numeric Rating Scale
WHO: World Health Organization:SD:Standard Deviation
MASCC/ISOO: Multinational Association of Supportive Care in Cancer and International Society of Oral Oncology
IRCT: Iranian Registry of Clinical Trials
SPSS: Statistical Package for Social Sciences
PKC: Protein kinase C
